# petal: Co-expression network modelling in R

**DOI:** 10.1186/s12918-016-0298-8

**Published:** 2016-08-01

**Authors:** Juli Petereit, Sebastian Smith, Frederick C. Harris, Karen A. Schlauch

**Affiliations:** University of Nevada, Reno, 1664 N. Virginia Street, Reno, 89557 USA

**Keywords:** Parameter-free algorithm, R, Small-world, Scale-free, Whole omics-approach

## Abstract

**Background:**

Networks provide effective models to study complex biological systems, such as gene and protein interaction networks. With the advent of new sequencing technologies, many life scientists are grasping for user-friendly methods and tools to examine biological components at the whole-systems level. Gene co-expression network analysis approaches are frequently used to successfully associate genes with biological processes and demonstrate great potential to gain further insights into the functionality of genes, thus becoming a standard approach in Systems Biology. Here the objective is to construct biologically meaningful and statistically strong co-expression networks, the identification of research dependent subnetworks, and the presentation of self-contained results.

**Results:**

We introduce petal, a novel approach to generate gene co-expression network models based on experimental gene expression measures. petal focuses on statistical, mathematical, and biological characteristics of both, input data and output network models. Often over-looked issues of current co-expression analysis tools include the assumption of data normality, which is seldom the case for hight-throughput expression data obtained from RNA-seq technologies. petal does not assume data normality, making it a statistically appropriate method for RNA-seq data. Also, network models are rarely tested for their known typical architecture: scale-free and small-world. petal explicitly constructs networks based on both these characteristics, thereby generating biologically meaningful models. Furthermore, many network analysis tools require a number of user-defined input variables, these often require tuning and/or an understanding of the underlying algorithm; petal requires no user input other than experimental data. This allows for reproducible results, and simplifies the use of petal. Lastly, this approach is specifically designed for very large high-throughput datasets; this way, petal’s network models represent as much of the entire system as possible to provide a whole-system approach.

**Conclusion:**

petal is a novel tool for generating co-expression network models of whole-genomics experiments. It is implemented in R and available as a library. Its application to several whole-genome experiments has generated novel meaningful results and has lead the way to new testing hypothesizes for further biological investigation.

**Electronic supplementary material:**

The online version of this article (doi:10.1186/s12918-016-0298-8) contains supplementary material, which is available to authorized users.

## Background

Within the life sciences, high-throughput technologies such as RNA-sequencing, microarrays, mass spectrometry, and ChIP-sequencing produce large experimental omics datasets at increasing volume. Analysts are left to organize, structure, and analyse these data in sufficient and efficient ways. Computational Biology, Bioinformatics, Systems Biology, Network Biology, and Network Medicine offer interdisciplinary tools to help solve these challenges. Here, our focus is the efficient and effective analysis of high-throughput gene expression data from microarrays and next-generation sequencing platforms (RNA-seq) via co-expression networks.

Applications of networks and their analysis have become standard tools in the Systems Biology toolbox for their versatility and powerful approach to whole-system analysis, their ability to handle very large complex datasets, and their proficiency to present large-scale gene association [1–4]. The networks can be examined with standard tools from Graph Theory to identify systematic changes, patterns, similarities and possibly regulations between genes. Co-expression network construction and analysis have found many uses in the life sciences, such as functional groupings of genes in plants under stress conditions, and identification of molecular targets for future targeted gene therapy [5, 6].

**Fig. 1:**
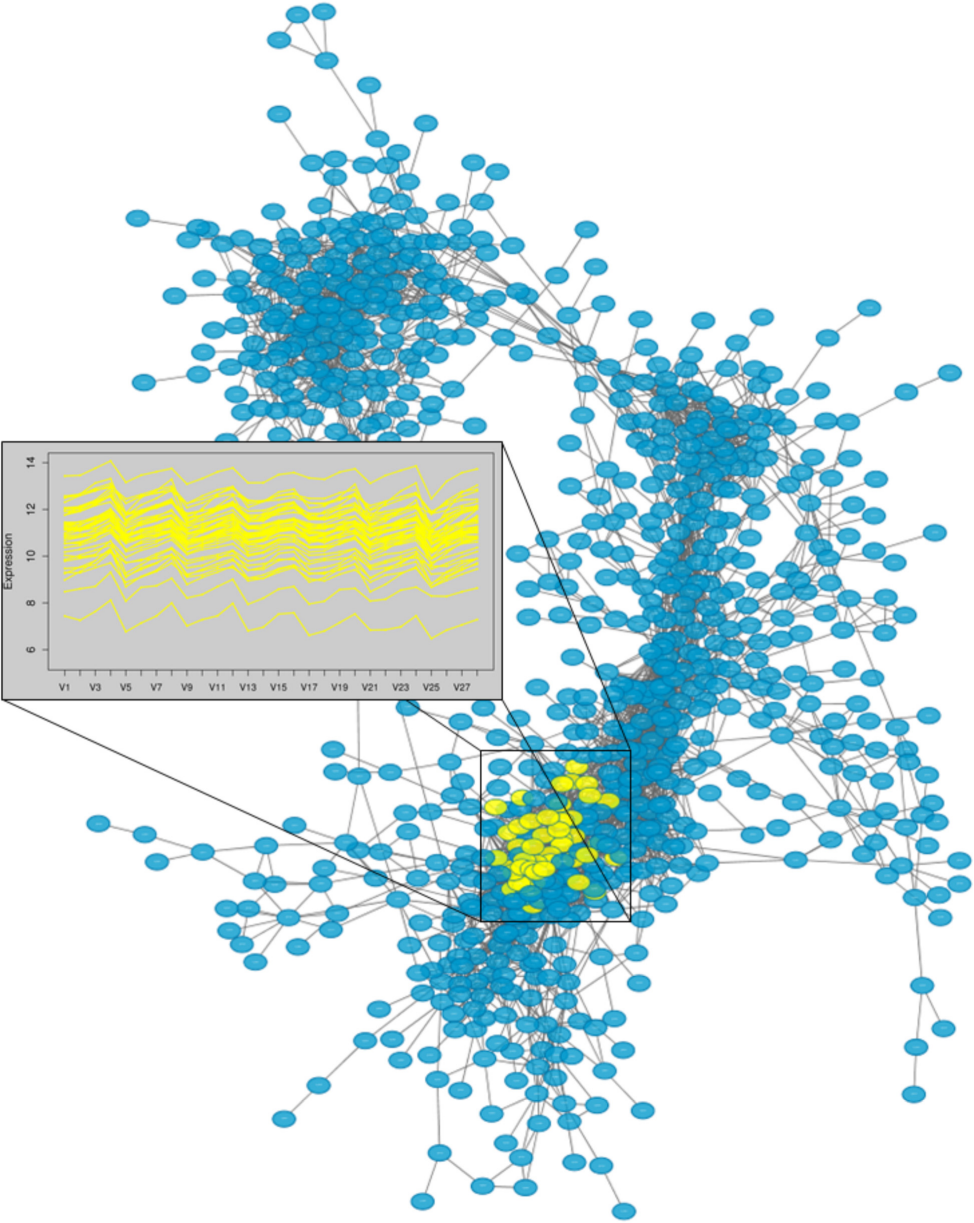
Sample network graph. *Blue* vertices (genes) are connected by an edge if a pre-defined association between vertices pairs is determined. A group of *yellow* vertices are highlighted, the genes corresponding to the yellow vertices have very similar expression profiles over 28 measurements

Co-expression networks are built from gene expression data collected over a series of experimental conditions, producing a data matrix of experimental expression measures of *m* gene across *n* conditions (treatments/time points/replicates). Vertices (nodes) correspond to genes; genes are connected by an edge if their expression measures across the *n* conditions are similar to a pre-defined degree. Figure 1 shows an example of a network graph and a highlighted group of genes with similar expression across 28 measures. Mathematically, the expression profile of a gene is an *n*-dimensional vector. Association between each gene pair (two *n*-dimensional vectors) is computed via an association measure, transforming the *m*×*n* expression matrix into an *m*×*m* symmetric association matrix.

Next, an adjacency function paired with a threshold transform the association measures into an unweighted or weighted network. In an unweighted network edges indicate only that an association exists between vertices implying a binary graph. In a weighted network all vertices are connected at different strength of association resulting in a completely connected graph. These networks, weighted or unweighted, are mathematically presented by the adjacency (incidence) matrix.

The resulting network model should follow typical properties of complex networks such as scale-free and small-world. Both these structural properties are standard characteristics of true complex biological network systems [7–13]. To determine these architectural characteristics of networks, topological measures taken from Graph Theory are calculated. These topological properties are robust descriptive measures that objectively describe the network’s architecture. Such measures include cluster coefficient, path-length, connectivity degree, vertices degree distribution, diameter, density, and many others [14].

**Small-world** In 1998 Duncan Watts and Steven Strogatz introduced a small-world network model [13]. For a network model to be small-world it must be made of densely connected subnetworks that are linked together in such a fashion that the path between any vertex pair is relatively short [13]. Mathematically, to categorize a network as small-world, its average cluster coefficient (meanCC) and average path length (meanPath) are calculated. A vertex’s cluster coefficient indicates how well its neighbours are connected: when a vertex has a cluster coefficient equal to one then all of its neighbours are connected to each other. In a small-world network model the average cluster coefficient of all vertices is larger than in a random graph. The path length between two vertices is the number of edges within their shortest path. The average path length of a small-world model must be relatively short in comparison to random network models. This phenomenon is often referred to as ‘six-degrees of separation’ [13, 15].

**Scale-free** Albert-László Barabási and Réka Albert inaugurated the notion of a scale-free network in 1999, and showed that most complex systems, including biological complex systems, are realistically modelled by networks following this property [10]. In a scale-free network, there are many vertices with few connections and only few vertices with a large number of connections. The degree of a vertex *i* is the number of connected neighbours of vertex *i*. Mathematically, a network is defined to have scale-free architecture when the degree distribution of the vertices follows a power-law distribution, *p*_*k*_, where *k* is the degree and *C* and *a* are positive constants [11, 12, 14]. The power-law function is shown in Eq. 1.

1 $$ p_{k} = Ck^{-a}  $$

After the network model is constructed it can be analysed. The underlying assumption of co-expression network analysis is that genes with similar expression patterns are possibly co-expressed, co-regulated, share common functionality, and/or might be regulated by a joint transcription factor. Consequently, groups of similar expression profiles across experimental conditions can be hypothesized to share common functionality by means of the ‘Guilt-by-Association’ principle [16]. As a result, common practice is to examine the constructed co-expression network for its topological properties to determine tightly connected vertices (clusters, modules) which help to reveal whole-system expression patterns, putative gene interactions, potential functional groupings, the association of functions to genes of unknown function, and possible regulations within the system. Examining network properties in combination with well-defined testing hypotheses can lead to the identification of putative key players within a pathway and thus possible drug targets in future research.

## Problem statement

We investigated multiple co-expression network applications and identified several challenges the life scientist might experience while using the applications:

1. The choice of a proper association measure relevant to data distribution and experimental hypothesis.
2. The absence of explicit confirmation that the constructed network follows the scale-free and small-world properties.
3. The inconvenience of having to enter a large number of user-specified input variables.
4. The restriction of using only datasets attached to a tool’s integrated database.
5. To know the meaning of gene modules.
6. To extract results of interest and/or interpreting the output presented by the application.

The measure used to transform the expression matrix into an association matrix should depend on the expression data distribution to be statistically valid. The Pearson Correlation Coefficient (PE) is the most common default measure in co-expression network tools [5, 17–21]. Cytoscape [22], a platform offering numerous network applications, has only one plugin that constructs co-expression networks simply based on PE. PE is a convenient choice because scientists are familiar with it, and its computational cost is very low in comparison to Spearman Correlation Coefficient (SP). On the contrary, PE is not an appropriate association measure for most data, as it is based on normality assumptions. For example, RNA-seq data typically follow a negative binomial distribution [23], hence PE is not a statistically robust measure and alternatives should be available.

Furthermore, co-expression networks are shown to have small-world and scale-free properties and can be realistically modelled by these two model structures [7–9]. To our knowledge there is no co-expression network method naturally constructing networks with both these biological properties. For example, Weighted Gene Co-expression Network Analysis (WGCNA) [24, 25], an R-library, includes a scale-free topological fitting index that can be manually tuned to construct a scale-free network, but WGCNA does not purposefully construct networks following small-world architecture, nor is the scale-free property robust within the WGCNA algorithm. A small change in user-specified parameters can shift the edge definition causing the loss of scale-freeness.

Another disadvantage of network analysis tools is that many methods assume that the user is familiar with network properties to select appropriate input variables or knows how to tune these variables. A common mandatory user-defined parameter is the association measure threshold. This threshold greatly affects conclusions drawn from the network model, as it governs the construction of the network. Therefore, the threshold should be objectively computed, rather than subjectively chosen by the user. Common practice is to define association with a Pearson Correlation Coefficient of 0.8 and greater [21, 26]. However, there is no consensus on threshold values; it is more of an arbitrary selection that does not necessarily reflect biological relevance.

Other network analysis tools are associated to particular databases or are organism-specific and can only generate network models from the integrated data [20, 27].

One of the main goals in network analysis is the identification of tightly connected subnetworks often referred to as gene modules: the general hypothesis is that genes with similar expression might also share functional similarity. Module detection is the general practice of defining (tightly) connected gene groups or partitioning the network into smaller subnetworks [14, 18, 25, 28]. Tightly connected groups are also called clusters or communities. The notion of ‘tightly connected’ has no consensus. The terms cluster, modules and communities are loosely defined and are interchangeably used in the literature [14, 25, 28]. In [5] gene modules are defined as disjoint subsets of nodes with more connections withhin the module than to genes outside the module. WGCNA labels modules as “clusters of nodes” and a “subset of nodes that are tightly connected to each other” [24]. Mahanta cursive state that “one of the most important applications of gene co-expression networks is to identify functional modules or network modules, which are represented by the strongly connected regions of the co-expression networks” [29]. Ficklin and Feltus describe genes in modules to “participate in similar biological processes; therefore, guilt-by-association inference can be applied to module genes with no known functions that are connected to module genes of known function” [30]. Modules identified by different methods inherit divergent graph properties. To obtain insights in regards to intra-connectivity within the extracted gene groups, their properties should be provided. For example, density can be calculated is a measure of tightness. Density is the proportion of all possible edges and edges actually present in the network model. A density of one implies that every vertex has an edge to every other vertex in the particular subnetwork. The closer the density is to 1, the more densely/tightly connected the subnetwork is.

Finally, networks are generated in order to study experimental hypotheses, thus resulting structures containing genes of interest (GoIs) should be extracted and presented to the user in a clear and understandable manner. Also, these results should be both self-contained and easily transferable to other standalone tools such as Cytoscape [22, 31] or Pajek [32].

## Implementation

The petal tool was designed to strengthen the standard flow of co-expression analysis. Upon the evaluation of current co-expression network tools [20, 25, 27, 33] our first goal was to develop a network construction algorithm confirming that resulting network models follow real biological network characteristics: scale-free and small-world [7–13]. An additional goal was to generate network models based on entire omics expression datasets to ensure a true whole-transcriptome or whole-genome representation rather than based on a pre-selected subset of genes (e.g., differentially expressed genes). Another main aim was to present the researcher with a tool that is easy to use and does not require any prior network theory knowledge. Consequently, the number of input parameters needed to be minimized.

**Fig. 2:**
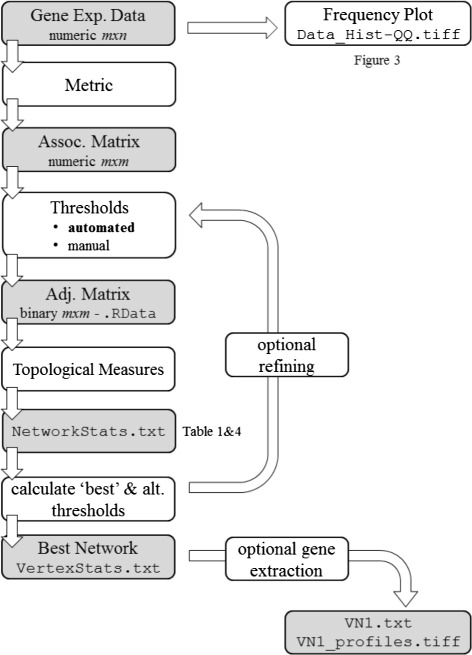
petal’s workflow. Illustration of computational pipeline implemented in R; *grey coloured rectangles* indicate data; *white rectangles* specify code; output files are writing in Courier New

The novelty of petal lies in its automated construction of scale-free and small-world network models. With no other user input but the experimental dataset, the construction of the network model is completely automated. The tool is implemented in the programming language R [34]. A summary of the computational pipeline is shown in Fig. 2 containing its main steps. In the following sections each step is discussed in more detail.

### Step 1: Defining association via a measure

**Fig. 3:**
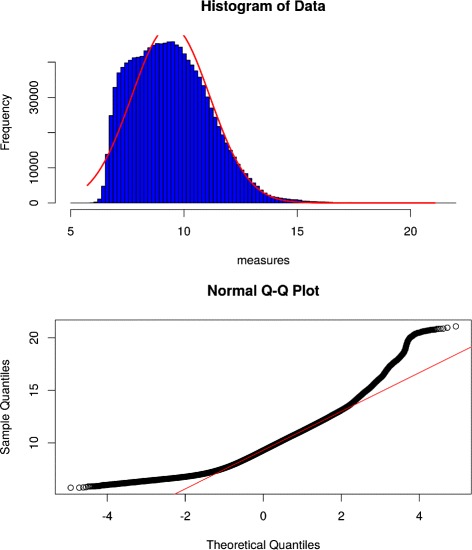
petal’s Histogram and Q-Q plot. Presentation of the output of graphHistQQFromFile

Metrics, such as measures of correlation or geometric distances, are applied to gene expression vectors to represent association between genes. petal includes the following measures: Pearson Correlation Coefficient (PE), Spearman Correlation Coefficient (SP), Kendall Rank Coefficient, Euclidean Distance, Manhattan Distance, Canberra Distance, and Mutual Information to account for parametric and non-parametric data distribution and to accommodate a variety of testing hypotheses. The default measure in petal is SP as it can be applied to non-normally distributed data. PE, a very popular measure [21, 26], is a parametric measure, and should only be applied to normally distributed (expression) data. A robust alternative to the PE is the Spearman Correlation Coefficient (SP) [35, 36]. PE, SP, and Kendall are correlation measures and compare the behaviour of the gene expression profiles (*n*-dimensional vector) over the *n* measurements. They solely evaluate the pattern similarity and do not calculate geometric distance between gene pairs, i.e., the spatial difference between gene expression vectors. Euclidean-, Manhattan-, and Canberra distances provide insight into the spacial difference between gene pairs, but do not provide any information in regards to common differential expression between gene pairs. Distances are non-parametric measures and can be applied to any data distribution. Mutual Information is another non-parametric measure based on entropy and can process missing data values better than other measures [37, 38]. Association can be calculated by a number of other less commonly used measures; for more detail on different measures refer to [18, 39–41].

petal offers an optional step to assist the user in deciding between a parametric and non-parametric measure based on their data distribution. petal provides a plotting function of the data’s histogram and the corresponding quantile-quantile plot (Q-Q plot). A histogram only demonstrates a rough presentation of the data’s distribution as it can be distressed by the number of considered bins. A normal curve based on boundaries of the input data is added to the histogram for easy comparison. To further ease the decision process, a Q-Q plot is added. A Q-Q plot is a mathematical approach to determine if data possibly arose from a theoretical distribution such as normal. The data at hand are compared to data generated from a normal distribution and plotted as a scatter plot. If the points roughly lie on a straight line, the distribution at hand can be considered normal. Both these methods are not proofs and only intent to help the user to examine their data to make an informed decision. The command graphHistQQFromFile(~myDataFile.txt~) pre-sents the user with a high resolution.tiff file, seen in Fig. 3. With this visual representation of the data, the user can determine a statistically appropriate measure. Note that SP can be applied to normally distributed data as well, but has less statistical power than PE, hence if the data is normal we recommend to use PE.

### Step 2: Defining edges via adjacency function and threshold

After the calculation of the association measures, they are transformed into an adjacency matrix according to a user-specified adjacency function and threshold. The simplest adjacency function is a discrete transformation that converts the expression association measures to 1 or 0 depending upon a user-selected threshold, to indicate similar expression or not, respectively. This transformation is called the Signum Adjacency Function [24] and is defined in Eq. 2, where the variable *α*_*ij*_ represents the association measure between gene *i* and gene *j* of the association matrix, *τ* is the pre-selected threshold on which to define association, i.e., an edge between vertices. Note that by definition in Eq. 2, the association measure *α* is a similarity measure, with highest possible numeric value indicating the strongest association. When *α* is a distance metric, the inequality signs in Eq. 2 are reversed.

2 $$ signum(\alpha_{ij}) = \left\{ \begin{array}{ll} 0 & \quad if ~~ \alpha_{ij} < \tau \\ 1 & \quad if ~~ \alpha_{ij} \geq \tau \end{array} \right.  $$

Unweighted network models have been widely studied in Graph Theory and carry well-defined properties. In consideration of these well established attributes, petal uses this discrete transformation (Eq. 2) to construct unweighted network models to take advantage of graph theoretical characteristics.

#### Calculating initial threshold list

The calculation of all pair-wise association measures of the *m*×*n* expression matrix results in *m*(*m*−1)/2 association measures, these are sorted from strongest to weakest association. For example, correlation and Mutual Information are organized in descending order, whereas distance measures are sorted in increasing order. For a network of *m* vertices to be connected, i.e., every vertex has a path to every other vertex in the network, it must have at least *m*−1 edges; thus the first threshold (*t*_*first*_), which is the most stringent, is set to the value at the (*m*−1)^*th*^ position in the sorted association measure list. The last threshold is based on several empirical evaluations: In a series of actual RNA-seq and microarray whole-omics test datasets, network models with edges more than 150 times the number of vertices prove to be too dense for evaluation within reasonable computational runtime. Furthermore, none of the observed cases of network models with this many edges could be classified as scale-free, their corresponding vertex degree distributions do not follow a power-law function. In consideration of both these empirical findings, we impose a restriction on all considered thresholds by limiting the number of edges to 150 times the number of vertices in the network. Consequently, the last threshold (*t*_*last*_) is set to the value at the (150×*m*)^*th*^ position in the sorted association measure list, see Table 1 for a visual representation.

**Table 1:** Sample sorted measure table used to define threshold list

Index	Gene1	Gene2	Measure value
1	*g* *e* *n* *e*_*u*_	*g* *e* *n* *e*_*w*_	1
2	*g* *e* *n* *e*_*q*_	*g* *e* *n* *e*_*r*_	0.98
⋮	⋮	⋮	⋮
*m*−1	*g* *e* *n* *e*_*t*_	*g* *e* *n* *e*_*q*_	*t*_*first*_
⋮	⋮	⋮	⋮
150×*m*	*g* *e* *n* *e*_*q*_	*g* *e* *n* *e*_*s*_	*t*_*last*_
⋮	⋮	⋮	⋮
*m*(*m*−1)/2	*g* *e* *n* *e*_*w*_	*g* *e* *n* *e*_*p*_	-1

Sorted measure table for correlation values ranging between [−1,1], value of 1 represents the strongest correlation. *m* is the number of genes; *p*,*q*,*r*,*s*,*t*,*u*,*w* are values within [ 1,*m*]; *t*_*first*_ and *t*_*last*_ are the first and last threshold values of the threshold list, respectively

The interval between first and last threshold is split into six equal subintervals. In Eq. 3 the step size, *Δt*, is calculated, which is then used to obtain a list of seven thresholds:

$$thresholds = (t_{first}, t_{first}+\Delta t, \dots, t_{first}+6\Delta t)$$

Based on empirical testing, the consideration of seven thresholds provide a sufficient spectrum of thresholds to construct networks models and determine scale-free and small-world. In the case in which first and last association threshold are far apart the width of subinterval could be relatively large. To accommodate for such a problem petal offers an optional step to refine the thresholds; this is described after in the section entitled “Refining threshold”.

3 $$ \frac{t_{first}-t_{last}}{6} = \Delta t  $$

#### Selection of ‘best’ threshold

Signum Adjacency Function (Eq. 2) is used with each threshold to generate adjacency matrices, each of which corresponds to a network model. The goal is to obtain a biologically and theoretically strong network model, thus the well-known properties of complex networks are imposed: small-world and scale-free. For each threshold a network model is constructed and its topological measures are calculated and reported, see Table 2, then each models’ measures are weighted against each other to determine the ‘best’ network model for downstream analysis.

**Table 2:** Sample network threshold table

thresh	*R* ^2^	slope/	mean	mean	%used	%bigComp
		power	CC	Path		
0.878	0.94	-2.02	0.36	6.74	21	32
0.834	0.93	-1.75	0.38	7.71	46	91
0.789	0.91	-1.58	0.40	5.70	68	97
0.745	0.87	-1.42	0.41	4.62	82	99
0.700	0.84	-1.26	0.42	3.91	91	99
0.656	0.78	-1.09	0.43	3.40	95	99
0.611	0.70	-0.92	0.44	3.02	98	99

Each row represents a network model based on the threshold in Column 1, *R* ^2^ and slope/power are are used to determine scale-free, meanCC and meanPath are used to conclude small-word, %used indicates how much of the original dataset is maintained, and %bigComp gives the percentage of vertices that are in the biggest component of the network model

##### Small-world

To evaluate a network model for small-world architecture, the average cluster coefficient and average path length are calculated, these are calculated for each network model and recorded as meanCC and meanPath, respectively (Column 4 and 5 in Table 2).

##### Scale-free

For each network model that petal generates, its actual degree distribution is calculated to evaluate if the model’s true degree distribution follows a power-law function (Eq. 1). A property of a power-law function is that its logarithmic transformation is linear in terms of log(*k*) as demonstrated in Eq. 4.

4 $$ \log(p_{k}) = -a \log(k)+ c  $$

With this linear transformation of the power-law function linear regression can be used. The true distribution is log-transformed. Linear regression is applied to the log-transformed degree distribution to determine the coefficient of determination (*R*^2^) and the slope of the linear regression. The slope *a* of the linear regression corresponds to the power in Eq. 1 and should lie within the interval (1,3) [12, 14]. When the linear regression is a good fit for the log-transformed true degree distribution, indicated by a high *R*^2^ value, and *a* in the interval (1,3) the model can be categorized as scale-free. petal evaluates each network model for how well the log-transformed degree distribution fits the a linear line via linear regression and records its corresponding *R*^2^ value and *a* to determine scale-free behaviour (Column 2 and 3 in Table 2).

##### Network components

A network component is a set of vertices that are connected by paths. If a network is made of one component it is considered a connected network. If a network model has two components, then this model has two disjoint subnetworks and not every vertex has a path to every other vertex within the entire network model. Network architectures, scale-free and small-world, are defined under the assumption that the network is connected; however, their defining topological properties (vertex degree distribution, average cluster coefficient, average path length) can be calculated without this assumption by excluding vertex pairs in different components when calculating averages. As a result, the calculated values for the network parameters can be very misleading if obtained from a disjoint network model. It is seldom the case for biological network models based on expression data to be one single component. The biggest component of a multi-component network must include at least 90–98 % of the network’s vertices for the topological measures to reliably define the model’s architecture, otherwise the topological measures can lead to misinterpretation [14]. Consequently, petal validates the reliability of the calculated network parameters (*R*^2^, *a*, meanCC, and meanPath) by determining the number of components in each model. This information is then used to identify the largest network component and its relative size to the entire current network model. To our knowledge, no other network construction algorithm considers the importance of verifying the percentage of genes in the largest component to uphold the calculated network characteristics. petal documents the relative size of the largest component (%bigComp) to confirm that the previously calculated properties as trustworthy (column 7 in Table 2).

##### Whole-genomics approach

One of our goals is to present a whole-omics approach and not just focus on a pre-selected set of genes. Therefore, an objective is to include as many genes as possible from the original dataset. For each network model, vertices which are not connected to any other vertex are removed from consideration as they do not provide any information in terms of association and the percentage of remaining vertices is recorded (column 6 in Table 2).

##### Weighting properties

The resulting network models are weighted against each other based on their topological properties. The ‘best’ threshold is considered to have generated a network model that is scale-free, small-world, with its biggest component including at least 95 % of the network’s vertices, and retains the maximum number of vertices from the original dataset. If such a network cannot be identified, the user is alerted that none of the considered network models are scale-free and small-world, but each model remains accessible.

#### Refining threshold

Depending on the calculated first and last thresholds, the interval between these two values can be relatively large. Consequently, the step sizes between considered thresholds are large and a ‘better’ threshold might be missed between the measured thresholds. To account for a large step size between threshold values, a refining step is included in the algorithm. Refining thresholds is an optional step, as this comes at a cost of longer runtime.

After the first round of initial threshold setting and identification of the ‘best’ threshold, it is not reported; instead, it is reused for a second round to test for scale-free and small-world. Let the ‘best’ threshold be denoted as *t*_*best*_. To calculate a new list of thresholds with smaller step size, new first and last thresholds are needed. We differentiate between two cases:

1. Besides *t*_*best*_, one or more thresholds also meet the criteria of the algorithm, denoted as *t*_*alt*_ for alternative thresholds. *t*_*alt*_ and *t*_*best*_ are sorted, the strongest and weakest associations are set to the new first and last thresholds, respectively.
2. Only *t*_*best*_ produces a scale-free, small-world network model. *t*_*best*−1_ and *t*_*best*+1_ are set to the first and last threshold, respectively.

With the assignment of the new first and last threshold, the interval between the two is again split into six equal subintervals, resulting in the list of refined thresholds. The new first and last thresholds cover a smaller spectrum resulting in smaller step sizes and thus making the choice of final threshold more precise. The algorithm then proceeds by recalculating the network threshold table.

### Step 3: Identifying structures within networks

One of the goals of co-expression network analysis is to extract structures (subnetworks, paths) from the entire network and examine these for biological patterns or association. Gene module detection is a standard procedure after network construction. Often hierarchical clustering is performed on a pairwise-distance matrix to organize the networks into hierarchical trees these can be cut at a user-specified height to obtain network modules. These modules can have very different topological properties. Furthermore, when modules are defined by hierarchical clustering some network information is lost, such as the interactions within a cluster (intra-connectivity).

#### Cliques

Cliques are completely connected subnetworks; every vertex connects to every other vertex. They share the same topological properties regardless of dimension. For example, the diameter, cluster coefficient, and density of any clique is always equal to one. The members of a clique form an equivalence class following the transitive property which results in less variation across clique members’ expression profiles compared to groupings obtained from standard clustering routines [25]. The mathematical definition of a clique is: A subnetwork of *j* vertices is a clique if and only if the subnetwork has *j*(*j*−1)/2 number of edges. Extracting cliques from a network is a common network analysis step, but computationally very expensive and an NP-complete problem.

Also, the extraction of fully connected subgraphs is considered too stringent for some biological testing hypotheses and very time-consuming when the network is densely connected. Another consideration when using cliques is that they might be too restricted in their properties based on the input data. If the input data are clean, meaning that technical or experimental noise and faulty measurements have been removed, then cliques are not considered stringent. Gene expression data is rather noisy for which cliques can be too inflexible of a structure. As an alternative fuzzy cliques can be used [18]. Fuzzy cliques are ‘almost’ cliques. Similar to modules, clusters, or communities, there is no standard definition of ‘almost’. When fuzzy cliques are discussed, topological properties should be reported to determine how strong or weak of fuzzy clique it is.

#### Extracting groups based on genes of interest

Another goal of co-expression network is to extract groups of genes that behave similarly over time or under varying environmental conditions. In general, the researcher has interest in a particular set of genes and wants to identify other genes which behave similarly to the genes of interest (GoIs). Consequently, petal allows the user to upload a list of gene identifiers to easily explore the GoIs. The genes of interest can be investigated more closely within the identified ‘best’ network by looking at their direct neighbourhoods referred to as vicinity networks (VN). A VN is a subnetwork representing the intermediate neighbourhood of a single vertex or of a completely connected set of vertices (clique). A VN of vertex *i* is a subnetwork including vertex *i* and all its direct neighbours and their edges. A VN of a clique includes all clique members and their common neighbours. Let there be *s* members in clique *r*, then the VN of clique *r* includes the *s* members and the the common neighbours of the *s* members. The topological properties of VNs can vary greatly, but their extraction from a network is very fast. These smaller subnetworks can be examined more closely and cliques are extracted at a much smaller computational cost from VNs than from the entire network. Often, some precision is lost when computational time is decreased; there is no loss of information when gene-specific cliques are extracted from its vicinity network than when they are mined from the entire network.

petal integrates two approaches to extract VNs:

1. Genes from the provided list are considered individually. For each entered gene a unique VN will be extracted and written to file. When this option is chosen, we recommend to keep the list of genes relatively short, such as twenty.
2. Assume *k* genes of interest were uploaded, to test for connections between these *k* genes, they are extracted from the network, resulting in a *k*×*k* adjacency matrix. From this subnetwork with *k* nodes all maximal cliques are identified. Each maximal clique is treated separately while identifying its neighbours. Neighbours of each maximal clique are written to a file distinguishing between neighbours and the clique genes obtained from the user’s identifiers.

If an annotation file is uploaded along with GoIs, the VN file will also include annotation. These output files are tab-delimited and can easily be manipulated or used without stand-alone analysis applications. Expression profiles for genes in each VN are graphed and saved as.tiff images. In addition, an analysis summary file is generated. Information in the summary file includes the number of genes loaded, followed by the number of genes which are not in the ‘best’ network model. Genes with no connections are removed, remaining genes are presented in a table format with their cluster coefficient and degree. Then each VN is listed and the gene of interest it includes. Lastly, a table containing the VN index, the size of each VN, the number of genes of interests (GoIs) it contains and its density is written to file. The density calculates how well the VN is connected. Table 3 shows a small example of this table. VN 54–56 can be considered fuzzy cliques, whereas VN 53 is not densely connected and requires a refined analysis.

**Table 3:** Sample vicinity network table

VN	VNsize	numGoI	density
1	2	1	1.00
2	36	1	0.53
3	8	1	0.82
4	20	1	0.52
⋮	⋮	⋮	⋮
52	24	2	0.72
53	63	2	0.68
54	17	3	0.86
55	10	4	0.93
56	27	5	0.89

Each row represents a particular vicinity network (VN). Column 1 shows the index of the VN, VNsize gives the number of vertices within the VN, numGoI is the number of genes of interest within the VN, and density indicates how well the VN is intra-connected

### petal’s main functions

petal is made of three main functions. dataToVNs requires two inputs: the file name of the expression data in tab-delimited format and the file containing the genes of interest (GoIs). This function takes the expression data matrix and supplies the user with groupings of the GoIs. Optional function input includes the upload of a gene annotation file, choice of measure, and thresholds. If no file name for the gene list is specified the function returns an error message and guides the user to use createSWSFnetFromFile. createSWSFnetFromFile only constructs the network models and calculates the ‘best’ model. This function can be followed up with downstreamAnalysis in which genes of interest (GoIs) can be loaded as well as a gene annotation file.

Usage:

dataToVNs(~myDataFile.txt~, ~myGenes.txt~,

~myGeneAnnotation.txt~)

createSWSFnetFromFile(~myDataFile.txt~)

downstreamAnalysis(winningThresh, metric,

~myGenes.txt~, ~myOutput.txt~,

~myDataFile.txt~,

~myGeneAnnotation.txt~)

### User Input

The user supplies the expression data file to petal. In addition, there are four optional steps: the selection of an association measure, user-specified thresholds (for the advanced user), the upload of a list of genes which are of particular interest to the researcher, and a gene annotation file. Additionally, the user has the option to evaluate their data distribution by using the function graphHistQQFromFile before constructing a network model. This provides the user with an estimate of their data distribution to identify whether data are approximately normally distributed. In this case, the user can then select a parametric similarity measure, such as the Pearson Correlation Coefficient (PE). The Spearman Correlation Coefficient (SP) is currently set as the default measure as it is data distribution-dependent. The second optional step is to select up to five association thresholds instead of using the automated threshold computation. To examine the network structures and association of a few genes petal allows the user to upload a list of genes which are extracted with their one-neighbour vicinity networks (VN) for in-depth evaluation. Lastly, if a gene annotation file is available, it can also be loaded in order to attach the information to each identified VN.

### User Output

Upon completion, petal’s accessible files include: general information file (.txt), network file (.txt), adjacency matrices (.RData), two topology tables (.txt), vicinity network files (.txt), and the expression profiles (.tiff) of each vicinity network. The network file can be directly uploaded into Cytoscape. Cytoscape, an Open Source tool, can be used for visualization and offers several network viewing tools via various plugins [22, 31, 42]. The.RData files of the network adjacency matrices are provided for convenient loading into R, enabling the advanced user to personalize downstream analysis if desired. In addition, the user can look at the characteristics of networks generated on different thresholds. Further, a table is provided which includes all network vertices with their degree and cluster coefficient. Each identified vicinity network is reported with gene membership and its density. Also each VN’s gene expression profiles can be viewed via.tiff image files.

## Results and discussion

### Key features

petal provides an easy to use R-library with the possibility of manual adjustment for the advanced user. With only one function call, the user obtains a sophisticated network analysis without any graph theoretical knowledge. The network model is guaranteed to be scale-free and small-world without any parameter specification. There is no tuning of parameters required. Gene specific groups can be extracted from the network which are conveniently automatically annotated if an annotation file is provided, and the expression profiles of all genes within the group are graphed. This feature saves the researcher a great amount of time. Furthermore, as the analysis can be done within one or two function calls, petal is accessible to scientists with minimal computer or R programming knowledge.

### Comparison to other tools

petal produces network models that present associations among genes of a studied system based on experimental data. These models provide a comprehensive view of the entire system which comes at a cost of longer computational runtime compared to most other current tools (e.g., WGCNA). On the other hand, user time is drastically reduced due to restricting user-intervention, decreasing the manual execution of computational steps. WGCNA, although very low in computational costs, does not purposefully generate small-world networks, and ensures scale-free networks only with user intervention. In addition, WGCNA’s extracts gene modules from a tree structure, which is a simplification of a network graph and information is lost in the process. WGCNA is a powerful network analysis tool, but requires many input parameters, making it hard for the novice user to take advantage of this R-library. Cytoscape [22, 31, 42] is a very popular tool to view networks. To our knowledge the construction of co-expression networks is unique to two plugins; one builds networks exclusively on the PE measure, and the other on the Mutual Information metric. petal offers a number of different measure and helps the user to choose an appropriate measure based on the specific expression data distribution. We also believe that petal’s output is very user-friendly so that the scientist can interpret the results easily and examine densely connected subnetworks of GoIs, both mathematically and via additional viewers such as Cytoscape or Pajek.

### Runtime and Memory

Mentioned in the previous section, petal has a longer runtime compare to other network analysis approaches because it constructs multiple network models to ensure the selection of a statistically appropriate and biologically relevant network model. The runtime depends on the individual dataset. Table 4 provides some guidelines based on empirical testing of a number of expression datasets generated on several platforms. It is evident that the calculation of PE is superior in speed to SP. The number of measurements does not have a notable influence on calculation time. The datasets of 15,137 genes with varying measurements finish in approximately the same time. The calculation of all pairwise association measures heavily affects the runtime in comparison to building individual network models, i.e., adjacency matrices.

**Table 4:** Empirical evaluation of petal’s runtime and memory requirement

Dimension of dataset	Metric	Runtime [hour]	Max memory [GB]
genes × measures			
5,000×7	PE	1.35	1.0
5,000×7	SP	2.07	1.0
11,342×16	PE	2.62	7.0
11,342×16	SP	4.42	7.5
15,137×12	SP	9.20	13.5
15,137×16	SP	9.13	15.0
15,137×28	SP	9.15	13.0

Each row is a separate run on a server with 2.5 GHz processors, of which petal used one and 256 GB RAM. Datasets of different sizes were supplied to createSWSFnet FromFile to monitor the runtime and memory usage of the function. In two runs PE was specified as the metric to demonstrate its fast computing time compare to SP: createSWSFnetFromFile(~myData.txt~, ~PE~)

### Application to the sciences

The utility of petal is demonstrated with an application of an Illumina RNA-seq whole-genome sequencing experiment of the mountain pine beetle (*Dendroctonus ponderosae*). Mountain pine beetles are obligate parasites of pine trees. They have destroyed a wide area of forest land and are a serious threat to conifer forests in the western North America. They rely on aggregation pheromones to coordinate the ‘mass attacks’ necessary to overwhelm a host tree’s defences and thus successfully colonize a tree. A molecular level understanding of this process may provide new methods to manage these devastating pests. Although pheromone biosynthetic pathways have been previously studied, the enzymes involved have not yet been completely identified, characterized, and understood [43–45]. Aw et al. presented the first genomic analysis of the mountain pine beetle and identified candidate genes encoding enzymes involved in pheromone-biosynthesis by studying their gene expression patterns [43], which yielded two confirmed pheromone biosynthesizing enzymes [46]. The hypothesis is that genes encoding these enzymes are regulated in parallel. Of particular interest is a group of 28 genes previously implicated in pheromone biosynthetic pathways.

#### Data

In this experiment, the Illumina NextSeq 500 platform was used to generate RNA-seq measures of gene transcription of more than 13,000 genes of the mountain pine beetle. Four biological replicates were collected for each of the four specimen types: fed/unfed male/female. Sequences were trimmed and filtered for nucleotide-base quality and 19–35 million sequences were aligned to the *Dendroctus ponderosae* reference genome. Unambiguously aligned sequences were counted for all annotated mountain pine beetle genes. Count data underwent standard protocols for low-count filtering, upper quartile normalization and transformation into counts per million following the DESeq2 processing pipeline [47]. Experimental findings relevant to beetle biology and biochemistry will be described in a forthcoming manuscript, in which the data will be made publicly available.

#### petal

After data quality control, the dataset contains 11,342 gene identifiers across 16 measures. The petal histogram and Q-Q plot confirms our assumption that our RNA-seq data are non-normally distributed (Additional file 1). The expression data are upload into petal alone with a list of 28 gene identifiers of interest and the corresponding gene annotation file. The 28 genes are analysed together as they have been hypothesized to play a joint role in the pheromone biosynthetic pathways. The petal run was performed on a server with 2.5 GHz processors and 256 GB RAM, one processor was used and it took 4.42 h utilizing at most 7.5 GB RAM.

#### Results

A series of seven thresholds ranging between 0.956 and 0.734 was determined based on SP measures of all pair-wise comparisons to generate a scale-free, small-world network. For all seven thresholds the adjacency matrices were generated and their topological properties calculated and presented in the NetworkStats.txt file (Table 5). Properties in Table 5 are used by petal to identify the ‘best’ threshold. The first column is the list of considered thresholds. The second and third columns represent the values obtained from the linear regression on the log-transformed degree distribution; meanCC is the mean cluster coefficient; meanPath is the average path length between vertex pairs; %used indicates the percentage of genes used from the original dataset signifying how many genes have connections within the specific network model; %bigComp describes how many of the network’s vertices are within the biggest component. petal identified a SP threshold of 0.808 to produce the ‘best’ scale-free, small-world network model. Inspecting Table 5, we see that thresholds above 0.845 are excluded from the decision process for ‘best’ threshold as the biggest component of those networks include less than 95 % of the network’s vertices and as a result the calculated properties are skewed by the high number of components. Also thresholds 0.771 and 0.734 are excluded due to their low coefficient of determination (*R*^2^). Consequently, only 0.808 and 0.845 remain; the network based on 0.808 contains 700 more genes than the model based on 0.845, thus providing a more whole-systems approach. As a result 0.808 is set to the ‘best’ network model and 0.845 is an alternative model.

**Table 5:** NetworkStats.txt obtained from petal for the mountain pine beetle dataset

thresh	*R* ^2^	slope/	mean	mean	%used	%bigComp
		power	CC	Path		
0.956	0.84	-1.71	0.44	6.89	21	22
0.919	0.90	-1.62	0.37	11.13	50	85
0.882	0.89	-1.45	0.38	7.19	72	94
0.845	0.86	-1.24	0.40	5.66	86	97
0.808	0.82	-1.05	0.42	4.71	94	99
0.771	0.77	-0.93	0.44	4.04	98	100
0.734	0.71	-0.85	0.47	3.55	99	100

Network parameters for each considered network model. Here, 0.808 constructs the ‘best’ network

**Fig. 4:**
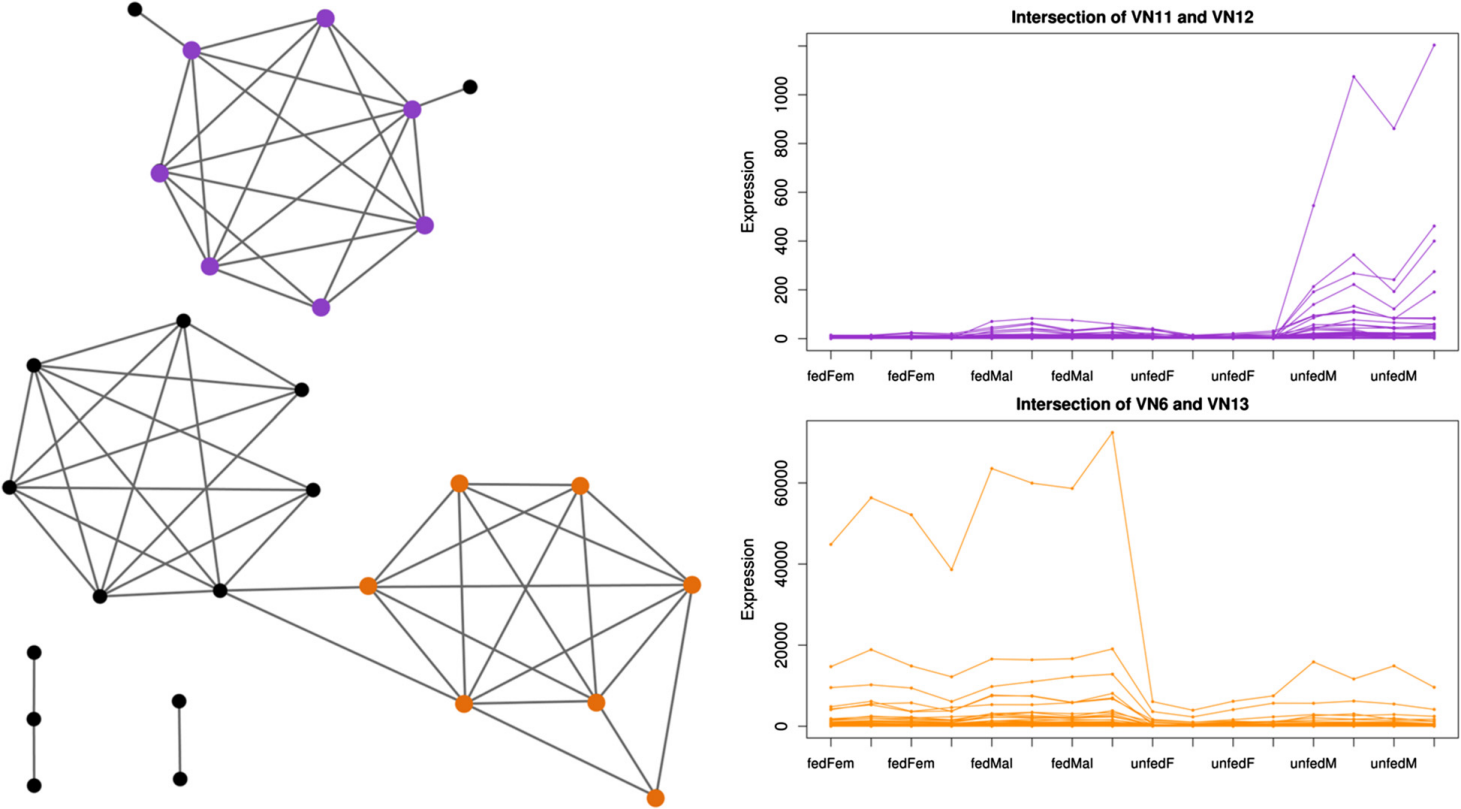
Subnetwork and grouped gene expression profiles. The subnetwork represents the 28 genes of interest extracted from a genomic network of the mountain pine beetle. Purple gene expression profiles are the intersection of VN11 and VN12. Orange gene expression profiles are the intersection of VN6 and VN13

The 28 GoIs and their edges are isolated from the 0.808 SP network model, which is presented in Fig. 4. This 28-vertices subnetwork has 13 maximal cliques, as a result 13 vicinity networks (VNs) are obtained (Additional file 2). Two of them, VN11 and VN12, are of special interest as they contain five and six of the 28 genes, with a density of 0.88 and 0.89, respectively. These two VNs overlap in four out of the 28 GoIs; overall these two VNs have a total intersection of 28 genes. The subnetwork of the 24 neighbour genes and the seven GoIs form a subnetwork with a density of 0.9871. The seven genes are highlighted in purple in Fig. 4. Because this 31 gene subnetwork is missing six edges to be a clique, we refer to this grouping as a fuzzy clique. The fuzzy clique’s gene expression profiles are shown in Fig. 4. The profiles indicate higher expression in male than in female mountain pine beetles. The expression difference is much more dramatic in the males which have not yet infested a tree and therefore have not eaten. This fuzzy clique is scientifically notable because some members encode enzymes with activities that are predicted to catalyse uncharacterised steps of synthesis in the pheromone component. Our analysis results are accordant with prior literature, the 31-node fuzzy clique identifies genes that encode enzymes already confirmed as pheromone biosynthetic enzymes. In addition, this fuzzy clique includes genes which previously have been predicted to catalyse known steps in the pheromone biosynthetic pathway. Within this identified grouping, the scientist is now able to narrow down targets for further wet lab examinations.

Another interesting subnetwork, VN6, is a vicinity network obtained from four other GoIs. All 85 common neighbours are tightly interconnected: the 89-gene subnetwork has a density of 0.96. Upon closer examination, VN6 and VN13 share three GoIs and their intersection includes 80 genes/vertices. Adding the four GoIs that do not overlap between the two VNs to the 80-gene intersection results in a subnetwork with a density of 0.9943. The union of the GoIs of VN6 and VN13 are highlighted in orange in Fig. 4 and the profiles are shown in orange. Biologically, this subnetwork presents a group of 84 very similarly expressed genes, including various cytochrome P450; this grouping agrees with a hypothesized link between tree resin detoxification and pheromone production [43, 46].

Overall, this approach enables the researcher to quickly view genes with similar expression patterns. With current annotation of the genes at hand, simple observations of the similarity or differences of functions of similarly-behaving genes can be made.

## Conclusion

petal is written for life scientists to construct high level co-expression networks and to extract vicinity networks of interest. petal is very user-friendly by requiring little prior knowledge of network science without sacrificing the quality output that comes from complex, well graph-theoretically defined networks. petal’s adaptability allows for the analysis of experimental expression data of most sizes. petal is an easy-to-use tool, attractive to a wide range of scientists with flexible and customizable options.

## Availability and requirements

**Project name:** petal**Project homepage:**https://github.com/julipetal/petalNet**Operating system:** Linux**Programming language:** R**Other requirements:** igraph (version 0.7)**License:** GPL-3**Restriction for non-academic use:** None

## Abbreviations

PE, pearson correlation coefficient; SP, spearman correlation coefficient; WGCNA, weighted gene co-expression network analysis; GoI, gene of interest; Q-Q plot, quantile-quantile plot; meanCC, mean cluster coefficient; meanPath, mean path length; VN, vicinity network

## Additional files

Additional file 1 petal’s Histogram and Q-Q plot of the Mountain Pine Beetle’s transformed expression data. (PDF 271 kb)

Additional file 2 petal’s output file from its genes of interest analysis. Genes of interests are listed with their corresponding cluster coefficient and degree, the number of maximal cliques within the gene of interest subnetwork is stated and each maximal clique’s members are provided with the associated vicinity network’s name, lastly a summary table of the vicinity networks is given. The summary table includes the name of the vicinity network (e.g. VN1, VN2), the number of nodes within the particular vicinity network (VNsize), the number of genes of interest within the vicinity network (GoInum), and its density. (TXT 196 kb)
