# Supplementary figures and images for: petal: Co-expression network modelling in R
Source: BMC Syst Biol. 2016 Aug 1;10(Suppl 2):51. doi: 10.1186/s12918-016-0298-8 (PMC4977474; doi:10.1186/s12918-016-0298-8)

# Histogram of Data

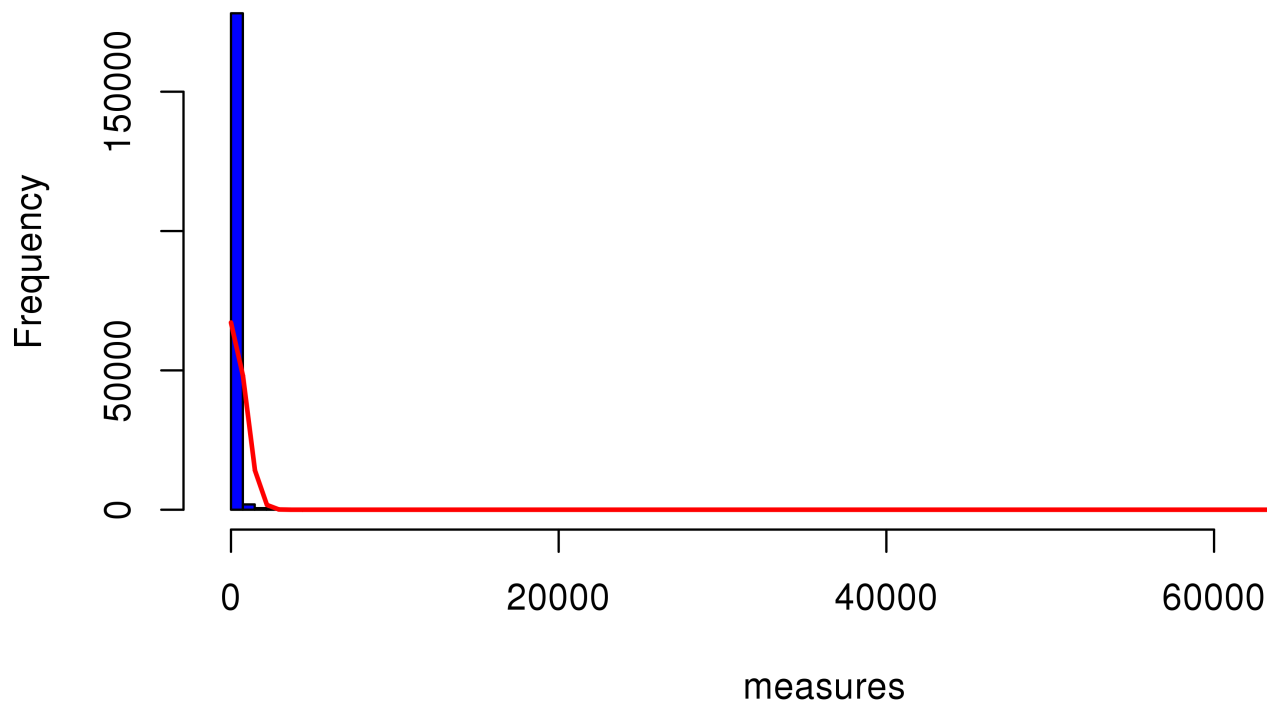

# Normal Q-Q Plot

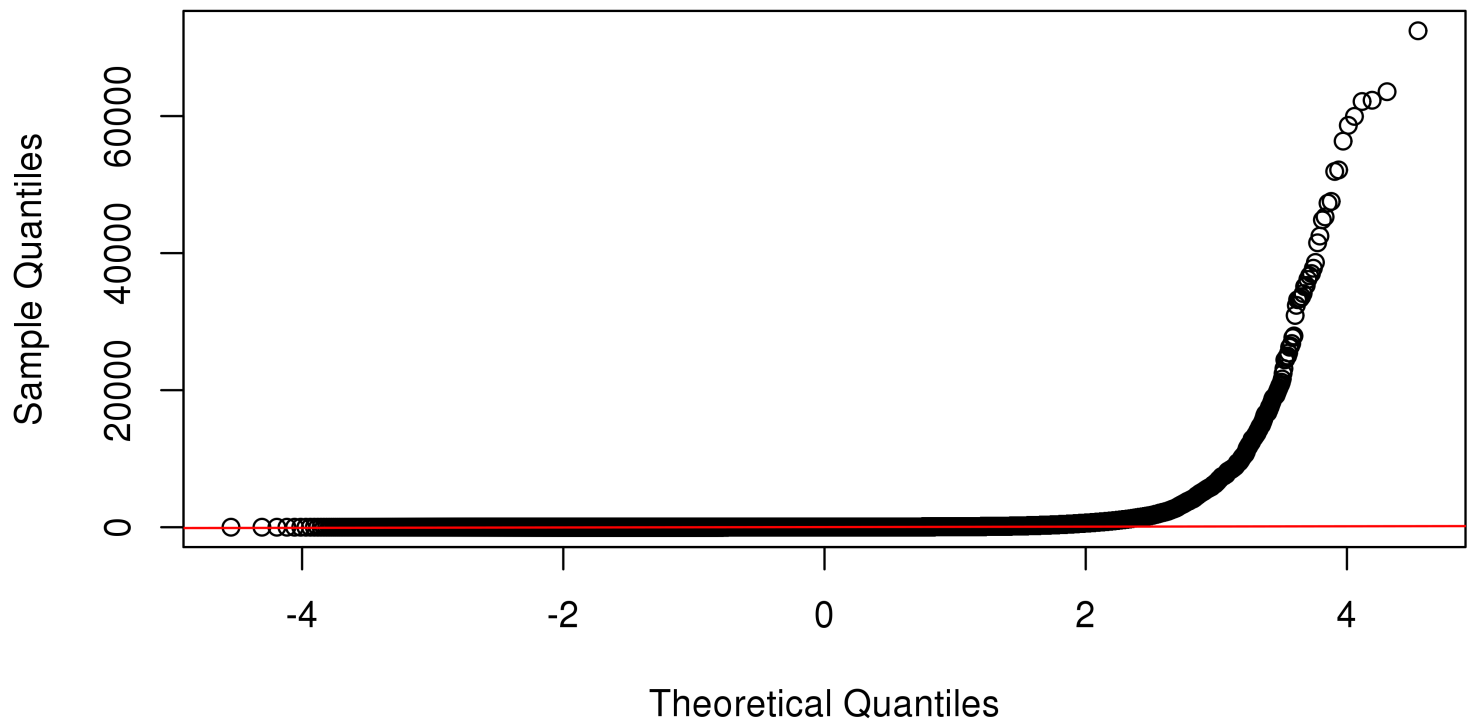

Supplement: Additional file 1 — petal’s Histogram and Q-Q plot of the Mountain Pine Beetle’s transformed expression data. (PDF 271 kb) [file 12918_2016_298_MOESM1_ESM.pdf]
